# PEX11B palmitoylation couples peroxisomal dysfunction with Schwann cells fail in diabetic neuropathy

**DOI:** 10.1186/s12929-024-01115-5

**Published:** 2025-02-12

**Authors:** Yu Mei Yang, Hang Bin Ma, Yue Xiong, Qian Wu, Xiu Kui Gao

**Affiliations:** 1 Department of Endocrinology, Center for Metabolism Medicine, the Fourth Affiliated Hospital of School of Medicine, and International School of Medicine, International Institutes of Medicine, Zhejiang University, Yiwu, 322000 China; 2 Department of Radiology, Center of Regenerative and Aging Medicine, the Fourth Affiliated Hospital of School of Medicine, and International School of Medicine, International Institutes of Medicine, Zhejiang University, Yiwu, 322000 China

**Keywords:** Diabetic neuropathy, Schwann cells, Peroxisomes, Palmitoylation, Mendelian randomization, Multiple sclerosis

## Abstract

**Background:**

Diabetic neuropathy (DN) is a prevalent and painful complication of diabetes; however, the mechanisms underlying its pathogenesis remain unclear, and effective clinical treatments are lacking. This study aims to explore the role of peroxisomes in Schwann cells in DN.

**Methods:**

The abundance of peroxisomes in the sciatic nerves of mice or Schwann cells was analyzed using laser confocal super-resolution imaging and western blotting. The RFP-GFP-SKL (Ser-Lys-Leu) probe was utilized to assess pexophagy (peroxisomes autophagy) levels. To evaluate the palmitoylation of PEX11B, the acyl-resin assisted capture (acyl-RAC) assay and the Acyl-Biotin Exchange (ABE) assay were employed. Additionally, MR (Mendelian randomization) analysis was conducted to investigate the potential causal relationship between DN and MS (Multiple sclerosis).

**Results:**

There was a decrease in peroxisomal abundance in the sciatic nerves of diabetic mice, and palmitic acid (PA) induced a reduction in peroxisomal abundance by inhibiting peroxisomal biogenesis in Schwann cells. Mechanistically, PA induced the palmitoylation of PEX11B at C25 site, disrupting its self-interaction and impeding peroxisome elongation. Fenofibrate, a PPARα agonist, effectively rescued peroxisomal dysfunction caused by PA and restored the peroxisomal abundance in diabetic mice. Lastly, MR analysis indicates a notable causal influence of DN on MS, with its onset and progression intricately linked to peroxisomal dysfunction.

**Conclusions:**

Targeting the peroxisomal biogenesis pathway may be an effective strategy for preventing and treating DN, underscoring the importance of addressing MS risk at the onset of DN.

**Supplementary Information:**

The online version contains supplementary material available at 10.1186/s12929-024-01115-5.

## Background

Diabetic neuropathy (DN) is one of the most common chronic complications of diabetes, with an estimated lifetime prevalence of 30–50% in people with diabetes [1–3]. It poses significant challenges, including an increased risk of mortality, foot complications such as lower limb amputations, severe pain, and a direct impact on daily functioning and quality of life [4]. With the persistent increase in diabetes prevalence and incidence, DN impacts medical care across a spectrum of providers, yet, to date, the pathogenic mechanisms of diabetic neuropathy remain poorly understood and no effective drugs are available [4, 5].

Demyelination and axonal loss are important clinical pathological features of DN [3, 6–8]. Schwann cells (SCs), as the principal cells of the peripheral nerve, play a crucial role in forming a multilayer myelin sheath around the axons of neurons [9, 10]. This myelin sheath enhances electrical impulse conduction, facilitates effective transmission, and safeguards neuronal axons [11, 12]. Additionally, the formation of the myelin sheath and maintenance of axonal energy homeostasis are closely related [13]. Notably, SCs demonstrate greater plasticity, retaining the ability to de-/transdifferentiate even after terminal differentiation [14]. This unique characteristic provides the peripheral nervous system with increased adaptability to stress, disease, and injury, resulting in remarkable resilience and the potential for regeneration and repair [14]. Growing evidence suggests that dysfunction of SCs plays a significant role in the pathogenesis of DN [10, 15–17]. Therefore, understanding the mechanisms underlying SCs dysfunction in DN and identifying effective therapeutic targets are of paramount importance in the prevention and treatment of DN.

Abnormal lipid metabolism is closely linked to the progression of DN [18–22]. Peroxisomes are single-membrane-bound organelles that are ubiquitous in eukaryotic cells, serving as multipurpose organelles involved in both catabolic and anabolic pathways, such as lipid metabolism, ether-phospholipid biosynthesis, and reactive oxygen species metabolism [23–25]. Of particular significance is their essential role in myelin formation and the normal function of SCs [26, 27]. Peroxisomes are crucial in creating and maintaining the lipid content of the myelin sheath, emphasizing their importance in the nervous system [27]. Mutations in genes related to peroxisomes can lead to peroxisomal biogenesis disorders (PBDs), a group of heterogeneous metabolic diseases characterized by impaired peroxisomal function [26]. Demyelination is also a key clinical pathological feature of PBDs [28]. Moreover, peroxisome dysregulation has been linked to various human disorders, including diabetes, multiple sclerosis (MS), and neurodegenerative diseases [26, 29]. Upregulation of catalase within peroxisomes, rather than mitochondria, has been shown to decrease oxidative stress and protect pancreatic β cells from palmitate-induced lipotoxicity [26]. Conversely, knockout of catalase in diabetic mice has been associated with increased oxidative stress and nephropathy symptoms [30]. However, the involvement of peroxisomes in SCs in the pathogenesis of DN remains unclear.

Protein palmitoylation is a reversible lipid modification that is mediated by a family of DHHC acyltransferases and can be reversed by a small group of acyl-protein thioesterases (APTs) [31, 32]. This post-translational modification impacts over 4,000 human proteins and has been largely underestimated as a potential factor in metabolic disorders [33]. Glucose has been observed to augment the palmitoylation of proteins in cellular models of pancreatic β cells [34]. Of particular importance, palmitic acid (PA), a high intake of which has been strongly linked to the development of various metabolic abnormalities like insulin resistance and type 2 diabetes [35–37], serves as a substrate for palmitoylation, suggesting that it may promote lipotoxicity through this process. Therefore, the palmitoylation associated with PA could play a significant role in the worsening of diabetic neuropathy. Recent research has demonstrated that increased levels of NLRP3 palmitoylation contribute to inflammatory responses in diabetic wounds [38], while a deficiency in APT1 has been linked to excessive insulin secretion, leading to β cell dysfunction, and mirroring the progression of certain forms of type 2 diabetes in humans [31]. However, the role of palmitoylation in diabetic neuropathy and its underlying molecular mechanisms still lack clarity.

Here, we have demonstrated a decrease in peroxisomal abundance in the sciatic nerve of diabetic mice. Further cellular-level analysis revealed that PA weakens peroxisomal biogenesis in SCs. Mechanistically, PA triggers the palmitoylation of PEX11B, an essential protein for peroxisomal fission, disrupting PEX11B self-interaction and hindering peroxisome fission. Remarkably, fenofibrate, a fibrate drug that acts as an agonist of peroxisome proliferator-activated receptor alpha (PPARα), showed remarkable efficacy in preventing PA-induced reductions in peroxisomal abundance and restoring the peroxisomal abundance in diabetic mice. Additionally, through mendelian randomization (MR) analysis, we found that DN can increase the risk of MS, indirectly indicating a close association between peroxisomal dysfunction and DN. These findings collectively unveil a mechanism by which palmitoylation regulates peroxisome biogenesis, resulting in SCs failure. This highlights the significant role of peroxisomal dysfunction in the development of DN and suggests that fenofibrate could be a promising therapeutic option for preventing and treating DN.

## Methods

### Antibodies and reagents

The following antibodies were used in the study: anti-PMP70 (Santa Cruz, sc-514728), anti-PMP70 (HUABIO, HA721969), anti-PEX11B (HUABIO, HA721124), anti-PEX16 (HUABIO, ER60976), anti-PEX19 (HUABIO, ET7110-89), anti-MBP (HUABIO, ET1702-15), anti-Cleaved-PARP (HUABIO, ET1608-10), anti-PEX5 (Proteintech, 12545-1-AP), anti-GFP (HUABIO, T1607-31), anti-Flag (MBL, M185-7), anti-Actin (HUABIO, EM21002), anti-Tubulin (Proteintech, 66009-1-lg), and PLIN2 (Proteintech, 15294-1-AP). Chemicals include Hoechst 33342 stain solution from Yeasen (40732ES03), palmitic acid (PA) from Siduorui Bio (PA01), oleic acid (OA) from Siduorui Bio (OA01), Fenofibrate from Aladdin (F129682), 2-BP from Medchemexpress (HY-111770) and Streptozotocin (STZ) from Sigma (S0130-1G).

### Diabetes induction and fenofibrate intervention

For the preparation of STZ solution, 10 mg of STZ is weighed and placed in aluminum-wrapped 2 ml tubes. A solution of sodium citrate is prepared by weighing 735 mg of sodium citrate, dissolving it in 49.5 ml of normal saline, and adjusting the pH to 4.5 using approximately 0.5 ml of 1 M citric acid to make 50 mM sodium citrate. The sodium citrate solution is then filtered with a 0.22 μm filter. The STZ is reconstituted by adding 1.428 ml of the 50 mM sodium citrate solution to each vial containing 10 mg of STZ to make a working solution right before injection. For the preparation of mice, the mice are fasted for 4 h prior to the experiment and their blood glucose is measured. 7.86 μL/g of the STZ working solution is injected via intraperitoneal injection. After injection, the mice are switched to a 10% w/v sucrose solution. This injection procedure is repeated four additional times over the next four days and blood glucose is measured weekly thereafter for 12 weeks.

For fenofibrate intervention, STZ mice were randomly divided into two groups: the vehicle group (Vehicle, water by gavage) and the fenofibrate group (Fenofibrate, 200 mg/kg/d by gavage). Fenofibrate was made into a suspension liquid (prepared every day) through vortex mixing with water before gavage and administered once daily for a 2-week intervention.

### Cell culture and transfection

RSC96 Schwann cells (Fenghui Bio, CL0476) at passage number range of 3–10 were cultured in DMEM medium containing 10% fetal bovine serum, 100 U/mL penicillin and 100 U/mL streptomycin. Cells in six-well plates were transfected with Lipo8000™ (Beyotime Bio, C0533) according to the manufacturer’s protocol. 48 h after transfection, the indicated detection was carried out. For longer-term effects of PA on Schwann cells, including more pronounced changes in protein abundance and cellular function, the PA treatment duration was 24 h. To assess early cellular responses, cells were exposed to PA for 6 or 12 h.

### Immunoprecipitation studies and western blot analyses

Immunoprecipitation was performed as previously described [39, 40]. Control cells or cells transfected with expression plasmids were lysed in lysis buffer (150 mM sodium chloride, 50 mM Tris, pH 7.3, 0.25 mM EDTA, 1% (w/v) sodium deoxycholate, 1% (v/v) Triton X-100, 0.2% sodium fluoride, 0.1% sodium orthovanadate, and protease inhibitor cocktail (Selleck Chemicals, B14001). Lysates were immunoprecipitated (IP) with anti-FLAG M2 beads (Bimake, B23102). Samples were run in SDS-PAGE gels and analyzed by western blotting with the indicated antibodies. Signal detection was performed using the Tanon 5200 imaging system.

### Detection of palmitoylated proteins

The acyl-RAC assay was conducted following a published procedure with slight modifications [41, 42]. In brief, cells were lysed at a concentration of 1 mg protein/mL in Buffer B (100 mM HEPES, pH 7.5, 1 mM EDTA, 2.5% SDS). Free thiol groups were blocked with 0.2% S-methyl methanethiosulfonate at 42 °C for 30 min. Proteins were precipitated with pre-chilled acetone at − 20 °C for 1 h or overnight, followed by three washes with 70% cold acetone and resuspension in 0.8 mL of Buffer C (100 mM HEPES, pH 7.5, 1 mM EDTA, 1% SDS). Samples were then divided into two tubes (0.3 mL each), mixed with 20 μL of PabPur SulfoLink Beads (Smart-Lifesciences Bio, SA018005), and incubated with either 0.4 M NH_2_OH or NaCl with constant rotation at room temperature for 3 h. The resins were washed with Buffer C containing 8 M urea five times (5 min each) at room temperature and then treated with SDS loading buffer at 99 °C for 10 min. Subsequently, samples were separated on SDS gels and subjected to western blot analysis.

The Acyl-Biotin Exchange (ABE) assay was meticulously performed according to a previously documented protocol, with minor adjustments [43, 44]. Initially, HEK293T cells were transfected 36 h prior with Flag-tagged PEX11B or its mutant counterparts and were then harvested and gently rinsed with cold PBS. Preceding cell lysis, N-ethylmaleimide (NEM) was solubilized in absolute ethanol and subsequently integrated into the lysis buffer (50 mM Tris–HCl pH 7.5, 150 mM NaCl, 1 mM MgCl_2_, 1% NP-40, 10% glycerol, and a protease inhibitor cocktail). The cell lysates were then subjected to sonication at 4 °C in the NEM-fortified lysis buffer, and the resultant supernatants were incubated with anti-Flag beads from Smart-Lifesciences (SA042005) at the same temperature for a duration of 2 h. Post-incubation, the beads underwent a rigorous washing regimen, consisting of five washes with lysis buffer set at pH 7.5, followed by three washes with lysis buffer adjusted to pH 7.2. Subsequently, the beads were resuspended in a freshly compounded lysis buffer enriched with hydroxylamine (HAM), which included 50 mM Tris–HCl (pH 7.2), 150 mM NaCl, 1 mM MgCl_2_, 1% NP-40, 10% glycerol, 1 M HAM, and the protease inhibitor. This treatment was conducted at room temperature for 1 h, followed by four washes with lysis buffer at pH 7.2 and three additional washes with lysis buffer adjusted to pH 6.2. The beads were then incubated with Biotin-BMCC at a concentration of 5 μM, in lysis buffer set at pH 6.2, at 4 ℃ for 1 h. This was succeeded by an extensive washing protocol, involving ten washes with lysis buffer at pH 6.2, and three final washes with lysis buffer adjusted to pH 7.5. The immunoprecipitated samples were subsequently analyzed via western blotting, streptavidin-HRP conjugated from Beyotime (A0303).

### Immunostaining

Immunostaining was performed as previously described [45]. Briefly, the sciatic nerves of mice were collected, fixed (4% PFA), and embedded to make sections (Leica CM1950). Following the sectioning, the sections were permeabilized with 0.2% TritonX-100 in PBS for 30 min at room temperature (RT). Subsequently, the slices were incubated with a blocking buffer consisting of 5% goat serum, 0.5% TritonX-100 in PBS for 1 h at RT. And then they were incubated with the primary antibody diluted in blocking buffer for 12 h at 4 °C or 3 h at RT. Tissue sections were washed in PBS before incubation with secondary antibody and hoechst staining for 1 h at RT. Immunolabeled sections were washed and mounted with FluorSave reagent (Merck).

### Fluorescence imaging

Images were acquired in *x*–*y*-*z* planes using a 100 × oil immersion objective with appropriate laser excitation on an IXplore SpinSR super resolution microscope (Olympus). For RFP-GFP-SKL imaging, the gain settings are adjusted to most of the RFP-GFP-SKL fluorescence in cells appeared as yellow puncta.

### Image analysis

The investigators were blinded to the treatment during data collection and analysis, and unblinding was conducted upon completion of the analysis for plotting. The number and area of peroxisomes were quantified utilizing QuPath 0.3.2. For line scan analysis, straight line across the peroxisomes were drawn as ROI, intensity of ROI from each channel were calculated by plot profile tool in Fiji (ImageJ). For co-localization analysis, Pearson’s correlation coefficient was calculated using Fiji. 

### Statistical analysis

Statistical analyses were performed using GraphPad Prism 8.0.2 (GraphPad Software, Inc.). The results are presented as mean ± SEM. Statistical significance was determined as denoted in the figure legends: *p < 0.05, **p < 0.01, ***p < 0.001, or ****p < 0.0001. When comparing between two groups, a two-tailed unpaired Student’s t-test was conducted if the data followed a normal distribution, and variances were similar based on an F-test (*p* > 0.05). Alternatively, a two-tailed unpaired Student’s t-test with Welch’s correction was used when variances were different via the F-test (*p* < 0.05). A Mann–Whitney test was performed if the data did not meet the criteria for normal distribution. For comparisons involving three or more groups, ANOVA followed by Dunnett’s post hoc test or Tukey’s post hoc test was applied.

### Mendelian randomization (MR) analysis

To assess causality between Diabetic Neuropathy (DN) and Multiple Sclerosis (MS), we performed a two-sample bidirectional MR, first using DN as a risk factor and MS as an outcome, then the reverse. The GWAS data for DN (finn-b-DM_NEUROPATHY) and MS (ebi-a-GCST003566) were obtained from the IEU OpenGWAS project (https://gwas.mrcieu.ac.uk/). Both populations are of European descent and include both males and females. The DN dataset consists of 162,201 controls and 1,415 cases, with a total of 16,380,195 SNPs. The MS dataset includes 10,395 controls and 4,888 cases, with a total of 7,910,365 SNPs.

From the GWAS summary data of DN, significant SNPs are selected based on the criteria of *p* < 5 × 10^–8^. A linkage disequilibrium coefficient of *r*^*2*^ is set to 0.001, with an LD region width of 10,000 kb to ensure independence among SNPs and to exclude the influence of genetic pleiotropy on the results. MS-related SNPs selected from the GWAS summary data of MS are extracted, with a minimum *r*^*2*^ > 0.8.

The causal relationship between exposure (DN) and outcome (MS) was verified using MR-Egger regression, Weight Median (WM), Inverse-Variance Weighted (IVW), Simple mode, and Weight model, with SNP as the instrumental variable. Additionally, heterogeneity analysis (Cochran Q test), pleiotropy testing, and sensitivity analysis (leave-one-out) were conducted. The aforementioned methods were implemented using the TwoSampleMR version 0.5.11 package in R 4.3.3 software.

## Results

### The peroxisomal abundance is significantly reduced in diabetic mice

**Fig. 1:**
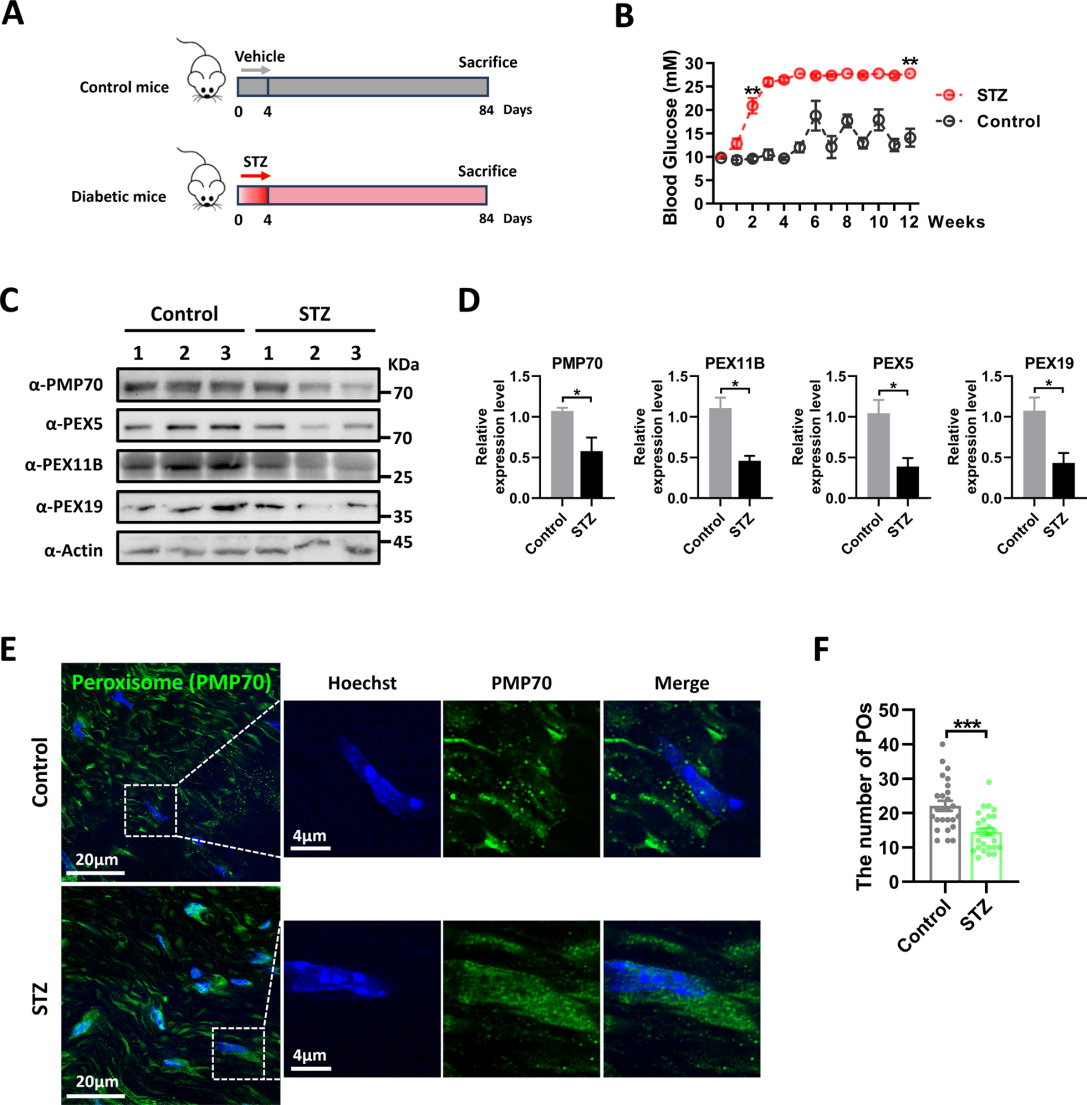
The abundance of peroxisomes in the sciatic nerves is decreased in diabetic mice. **A** A schematic diagram showing the treatments of the mice. **B** Blood glucose monitoring. Data is shown as means ± SEM with two-way ANOVA with Sidak’s tests (n = 8 per group). **p < 0.01. **C** Immunoblotting with antibodies against peroxisomes-related proteins in sciatic nerves from control and STZ-induced diabetic mice (n = 3 per group). Actin was used as a loading control. **D** Quantification of peroxisomes-related proteins. Data is shown as means ± SEM with Unpaired t test. *p < 0.05. **E** Representative immunofluorescence staining for PMP70 (green) on sciatic nerves sections from control and STZ-induced diabetic mice. Right: enlarged image of area indicated by dashed box. POs: peroxisomes. **F** Quantification of peroxisomes (POs) number. Data is shown as means ± SD with Unpaired t test (n = 26 cells). ***p < 0.001

To investigate the role of peroxisomes in diabetic neuropathy (DN), we induced diabetes in mice by administering low doses of streptozotocin (STZ) injections four times (Fig. 1A). In the second week, blood glucose levels were significantly elevated in STZ-induced diabetic mice (20.9 mM) compared to normal control mice (9.6 mM) and reached the upper limit (27.8 mM) of glucose detection by the fifth week, maintaining a high blood glucose state (Fig. 1B), confirming the successful establishment of the model. After 84 days, sciatic nerves were collected for analysis using western blotting and immunofluorescence. We observed a notable decrease in the expression of peroxisomal proteins (PMP70, PEX11B, PEX5, and PEX19) in the sciatic nerves of diabetic mice (Fig. 1C, D). Additionally, using laser confocal super-resolution imaging, we observed a significant reduction in the number of peroxisomes in the SCs of the sciatic nerves in diabetic mice by PMP70 staining (Fig. 1E, F). These findings suggest a decrease in peroxisome abundance within the sciatic nerves of diabetic mice.

### PA induces lipotoxicity and reduces peroxisomal abundance in Schwann cells

**Fig. 2:**
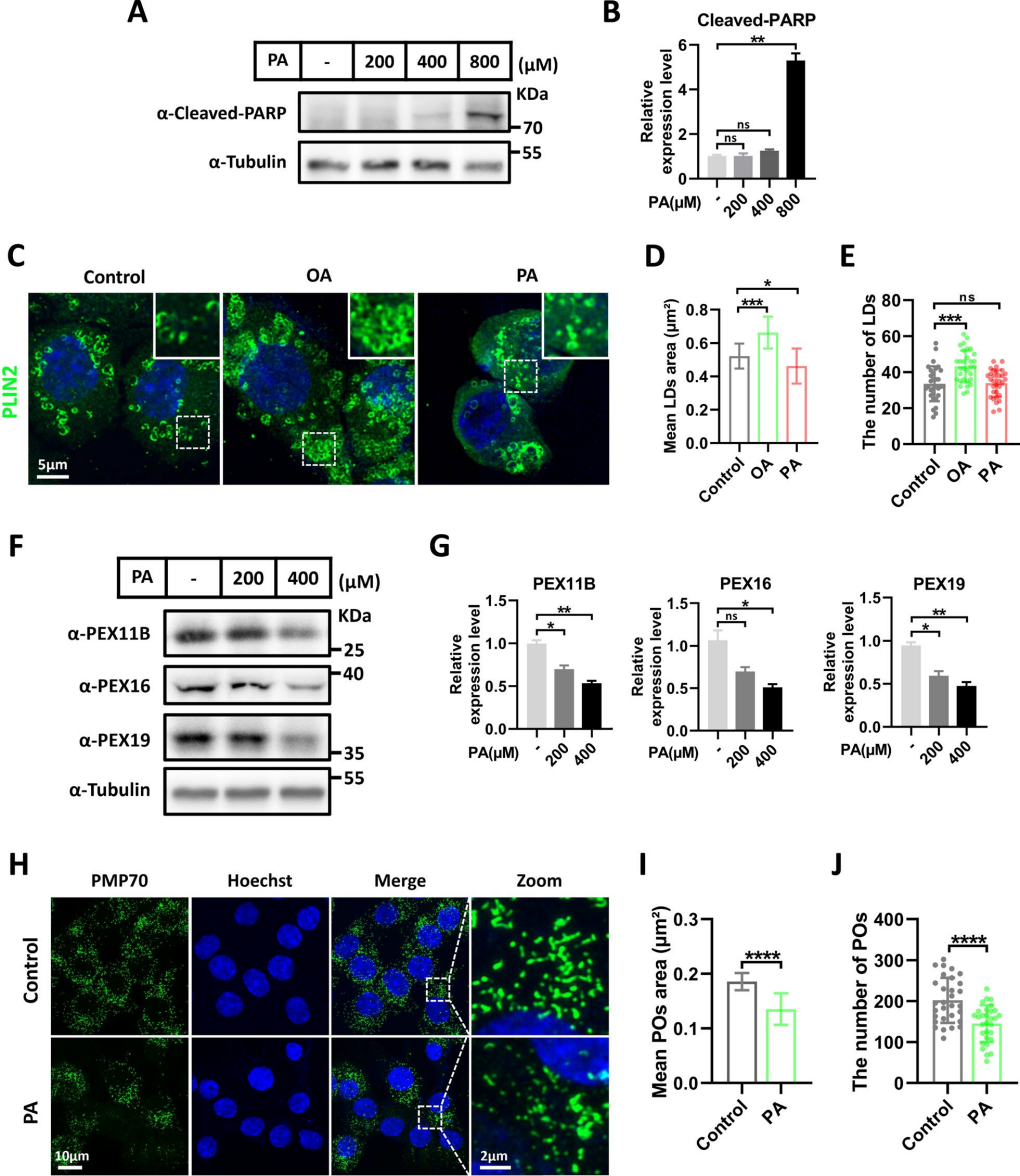
PA-induced lipotoxicity leads to a decrease in peroxisome abundance in Schwann cells. **A** Western blot detection of cleaved-PARP in Schwann cells (RSC96) treated with the different concentrations of PA. Tubulin was used as a loading control. **B** Quantification of cleaved-PARP protein. Data is shown as means ± SEM with Unpaired t test. ns, no significance. **p < 0.01. **C** Representative immunofluorescence staining for Plin2 (green) in RSC96 cells treated with 200 μM OA and 200 μM PA for 24 h. **D** Quantification of lipid droplets (LDs) area. Data is shown as means ± SD with One-way ANOVA Dunnett’s test (n = 31 cells). *p < 0.05, ***p < 0.001. **E** Quantification of lipid droplets (LDs) number. Data is shown as means ± SD with One-way ANOVA Dunnett’s test (n = 31 cells). ns, no significance. ***p < 0.001. **F** Western blot detection of peroxisomes-related proteins in RSC96 cells treated with different concentrations of PA. Tubulin was used as a loading control. **G** Quantification of peroxisomes-related proteins. Data is shown as means ± SEM with Brown-Forsythe and Welch ANOVA test. ns, no significance. *p < 0.05, **p < 0.01. **H** Representative immunofluorescence staining for PMP70 (green) in RSC96 cells treated with 400 μM PA for 24 h. Zoom: enlarged image of area indicated by dashed box. **I** Quantification of peroxisomes (POs) area. Data is shown as means ± SD with Unpaired t test (n = 30 cells). ****p < 0.0001. **J** Quantification of peroxisomes (POs) number. Data is shown as means ± SD with Unpaired t test (n = 30 cells). ****p < 0.0001

PA exposure inducing lipotoxicity is implicated in the pathogenesis of type 2 diabetes [35, 36, 46]. In order to elucidate the molecular mechanisms underlying peroxisomal dysregulation in SCs in DN, we treated RSC96 Schwann cells with PA to mimic the damage of SCs under pathological insulin resistance conditions. Firstly, we determined the appropriate induction concentration by treating RSC96 cells with different doses (0, 0.2, 0.4, and 0.8 mM) of PA (Fig. 2A). Through protein immunoblotting experiments, we observed that 0.8 mM PA treatment significantly elevated cleaved-PARP protein levels, indicative of cell apoptosis, whereas lower concentrations of 0.2 and 0.4 mM did not induce apoptosis (Fig. 2A). Additionally, using the lipid droplet marker protein PLIN2 staining, we observed that oleic acid (OA) treatment significantly increased the number and size of lipid droplets, while 0.4 mM PA treatment resulted in a decrease in the area and a slight decrease in the number of lipid droplets (Fig. 2C–E), consistent with the reported lipotoxicity of PA [47]. Building upon this, we found that PA treatment dose-dependently reduced the protein levels of peroxisomal proteins (PEX11B, PEX16, and PEX19) (Fig. 2F, G). Furthermore, super-resolution confocal microscopy imaging revealed a significant reduction in the number and size of peroxisomes with PA treatment (Fig. 2H–J). Moreover, time-course experiments showed that PA treatment reduced peroxisomes protein abundance in a time-dependent manner (Supplemental Fig. 1A, B). These experiments indicate that PA can diminish the abundance of peroxisomes in RSC96 cells.

### Long-time PA exposure inhibits pexophagy

**Fig. 3:**
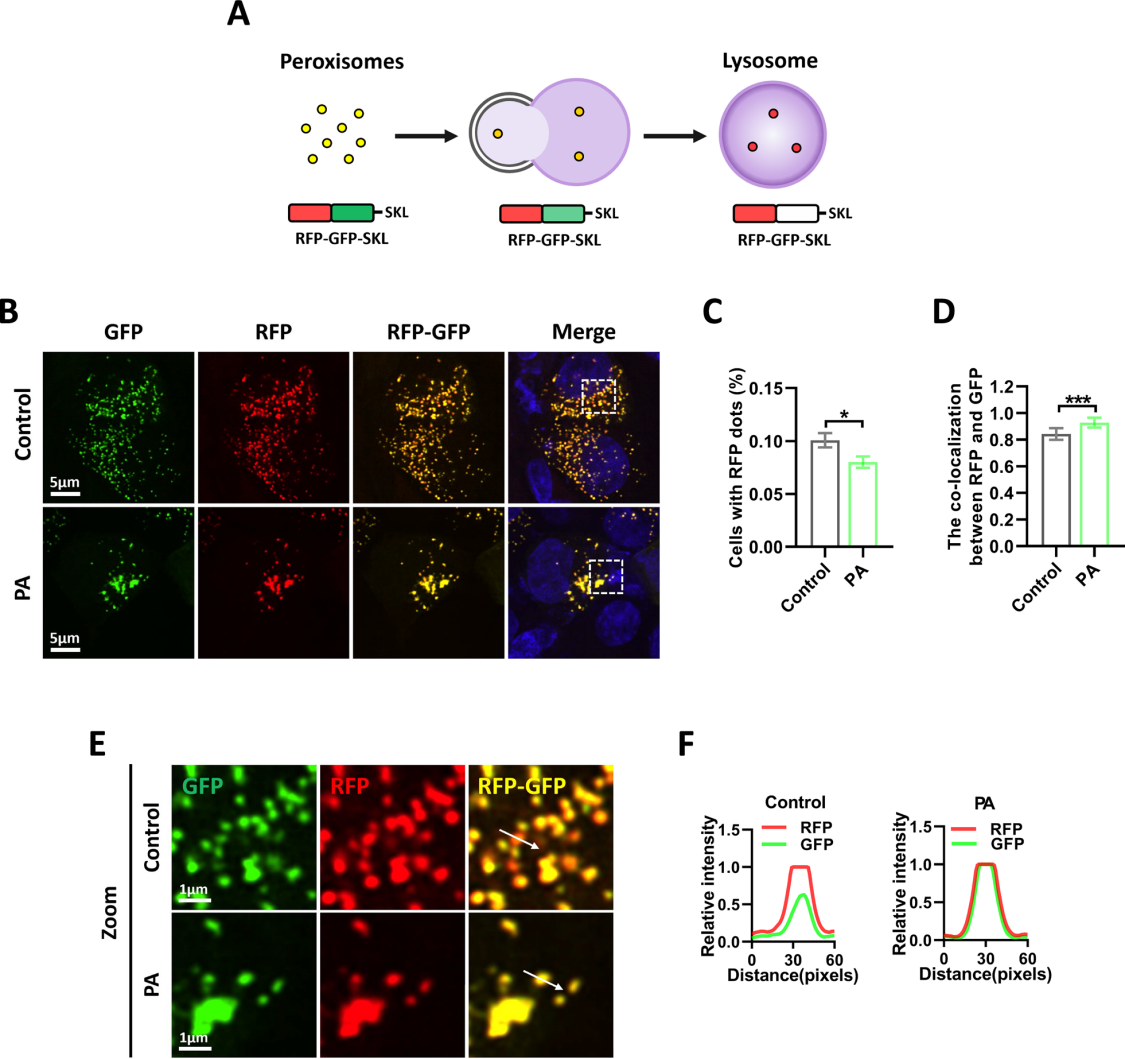
Long-time PA exposure inhibits pexophagy. **A** Schematic representation of the RFP-GFP-SKL (pexophagy probe) color change. **B** Representative fluorescence image of RFP-GFP-SKL in RSC96 cells treated with 400 μM PA for 24 h. **C** Quantification of red puncta from (**B**). Data is shown as means ± SEM with Unpaired t test (n = 24 cells). *p < 0.05. **D** Pearson’s correlation coefficient for co-localization between RFP and GFP was calculated from (**B**). Data is shown as means ± SD with Unpaired t test (n = 24 cells). ***p < 0.001. **E** Zoom: enlarged image of area indicated by dashed box from (**B**). **F** Plot profile from (**E**) showing the relative intensity of GFP and RFP

The maintenance of peroxisome abundance is primarily regulated by a delicate equilibrium between peroxisome biogenesis and degradation [48]. Peroxisomal degradation predominantly occurs through selective autophagy, specifically pexophagy [23, 49, 50]. Pexophagy not only helps reduce excess peroxisomes but also clears damaged peroxisomes, thereby improving the quality of peroxisomes [50]. We first examined whether PA affects pexophagy. To assess the impact of PA on pexophagy, a pexophagy probe was developed using a tandem fluorescently labeled RFP-GFP fusion protein fused with the peroxisome targeting signal Ser-Lys-Leu (SKL) (Fig. 3A), which facilitates the transport of matrix proteins destined for peroxisomes, enabling a direct assessment of pexophagy levels [51]. GFP fluorescence is quenched under the acidic conditions of autolysosomes, distinguishing intact peroxisomes as yellow puncta, and indicating pexophagy with single red puncta (Fig. 3A). Considering some peroxisomal proteins began to downregulate after 6 h of PA exposure (Supplemental Fig. 1A and B), we chose 6 h to treat the cells with PA. We found that PA did not alter the proportion of red puncta (Supplemental Fig. 1C and D), suggesting unchanged pexophagy. This data indicated that the PA-induced decrease in peroxisome abundance was not a result of its impact on peroxisome degradation. Given that multiple peroxisomal proteins serve as pexophagy receptors [50], we further asked whether long-time PA treatment inhibits pexophagy. Upon extending the PA treatment to 24 h, we observed a reduction in the proportion of red puncta (Fig. 3B, C, E). Moreover, the co-localization of RFP and GFP signals also significantly increased (Fig. 3D–F). These results indicate that long-time PA exposure inhibits pexophagy, which may exacerbate peroxisomal dysfunction. Therefore, we proposed that the inhibitory effect of PA on peroxisomes abundance is primarily due to its impact on peroxisome biogenesis rather than affecting peroxisomes degradation.

### Inhibiting palmitoylation rescues the reduction in peroxisomal abundance induced by PA

**Fig. 4.**
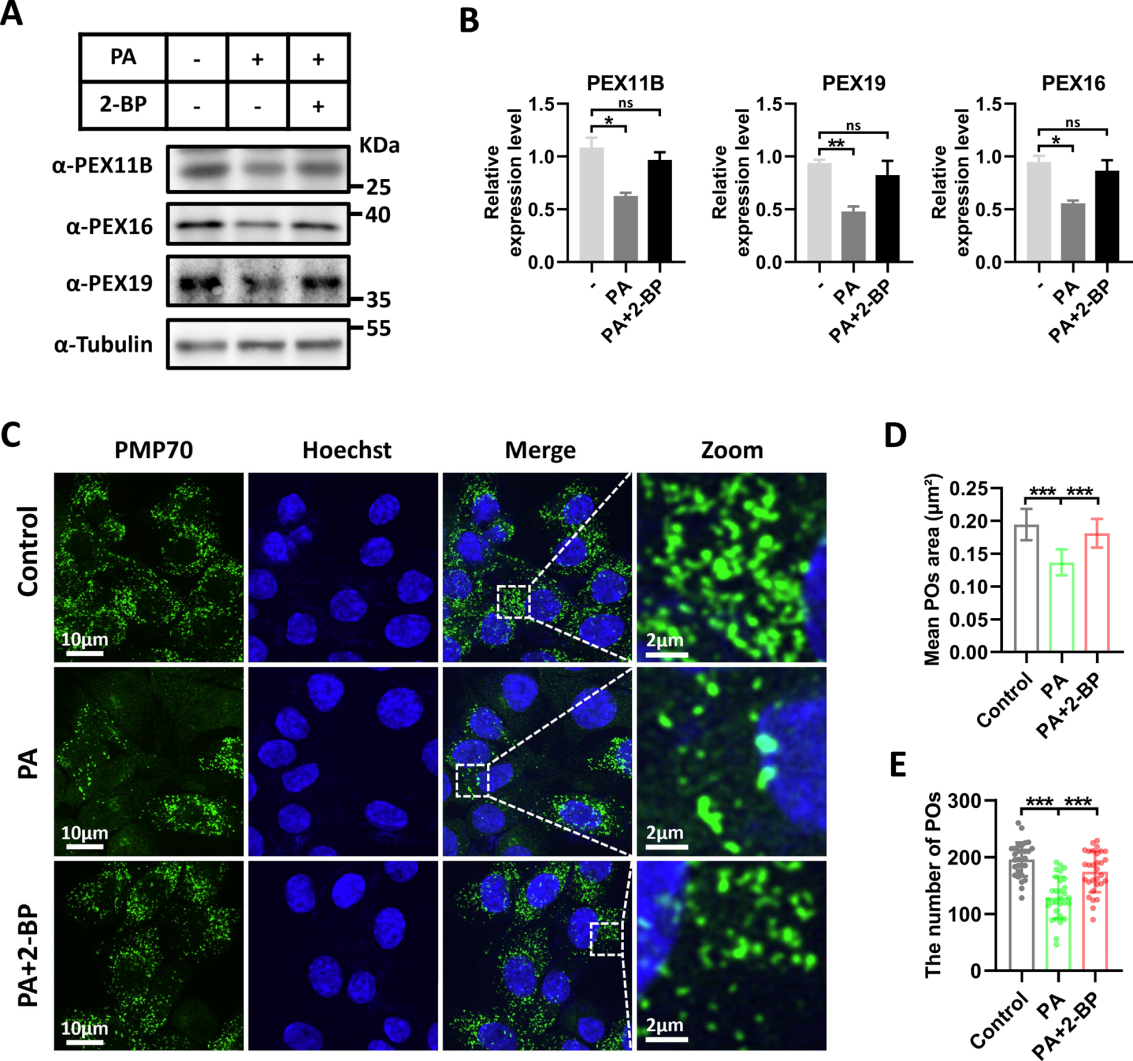
2-BP treatment rescues the reduction in peroxisomal abundance induced by PA. **A** Western blot detection of peroxisomes-related proteins under PA (400 μM) and PA (400 μM) with 2-BP (50 μM) conditions for 24 h in RSC96 cells. Tubulin was used as a loading control. **B** Quantification of peroxisomes-related proteins. Data is shown as means ± SEM with One-way ANOVA Tukey's test (n = 3 independent experiments). ns, no significance. *p < 0.05, **p < 0.01. **C** Representative immunofluorescence staining for PMP70 (green) under PA (400 μM) and PA (400 μM) with 2-BP (50 μM) conditions for 24 h in RSC96 cells. Zoom: enlarged image of area indicated by dashed box. **D** Quantification of peroxisomes (POs) area. Data is shown as means ± SD with One-way ANOVA Dunnett’s test (n = 30 cells). ***p < 0.001. **E** Quantification of peroxisomes (POs) number. Data is shown as means ± SD with One-way ANOVA Dunnett's test (n = 30 cells). ns, no significance. ***p < 0.001

We then explored how PA regulates peroxisome biogenesis. Palmitoylation is closely associated with diabetes and its complications [31, 32, 38], and PA is a substrate for palmitoylation. Therefore, we hypothesized that PA-mediated palmitoylation may regulate peroxisome fission. To test this hypothesis, we employed the palmitoylation inhibitor 2-BP and found that it rescued the protein levels of peroxisomal proteins (PEX11B, PEX16, and PEX19) (Fig. 4A, B). Additionally, 2-BP was also able to restore peroxisome abundance (Fig. 4C–E). These results suggest that palmitoylation regulates peroxisome fission.

### PEX11B undergoes palmitoylation at C25 site, which inhibits its self-interaction and peroxisomal elongations

**Fig. 5:**
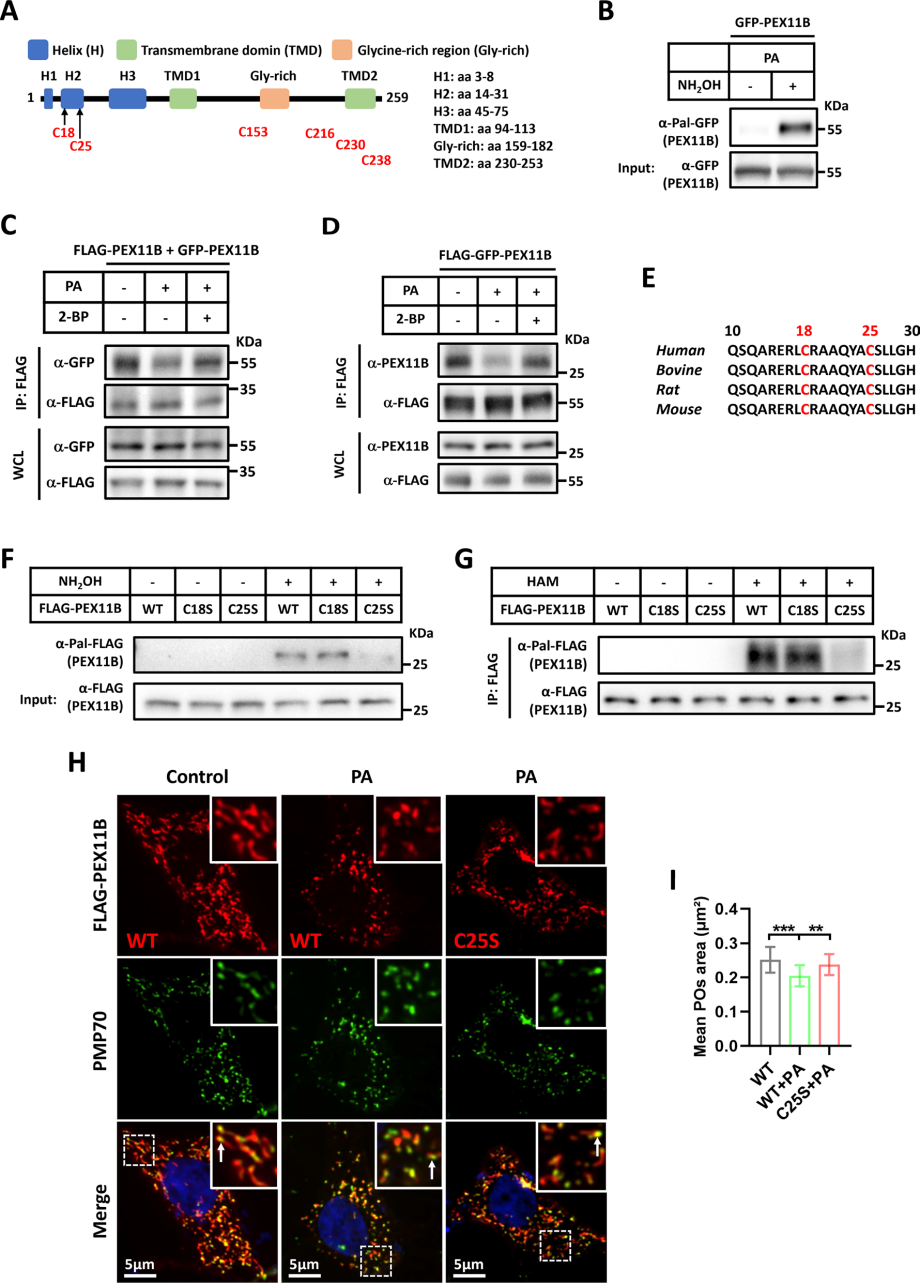
PEX11B undergoes palmitoylation at C25 site, which impairs its function. **A** Schematic diagram of PEX11B and its predicted palmitoylation sites. **B** RSC96 cells were transfected with GFP-PEX11B, followed by PA treatment for 12 h. Cells were harvested for palmitoylation assay. **C** RSC96 cells were co-transfected with FLAG- PEX11B and GFP-PEX11B, followed by PA (400 μM) or PA (400 μM) with 2-BP (50 μM) treatment for 6 h. Cell lysates were collected for immunoprecipitation (IP) and immunoblot analysis to detect the GFP-PEX11B. WCL, whole-cell lysates. **D** RSC96 cells were transfected with FLAG-GFP-PEX11B, followed by PA (400 μM) or PA (400 μM) with 2-BP (50 μM) treatment for 6 h. Cell lysates were collected for immunoprecipitation (IP) and immunoblot analysis to detect the endogenous PEX11B. WCL, whole-cell lysates. **E** Alignment of PEX11B sequences containing potential palmitoylation sites of different species. **F** 293 T cells were transfected with FLAG-PEX11B WT, C18S or C25S respectively. Cells were harvested for palmitoylation assay with acyl-RAC method. **G** 293 T cells were transfected with FLAG-PEX11B WT, C18S and C25S respectively. Cells were harvested for palmitoylation assay with ABE method. **H** Representative immunofluorescence staining for FLAG-PEX11B (Red) and PMP70 (green) in RSC96 cells. Zoom: enlarged image of area indicated by dashed box. **I** Quantification of peroxisomes (POs) area. Data is shown as means ± SD with One-way ANOVA Dunnett’s test (n = 31 cells). ***p < 0.001, **p < 0.01

Peroxisome fission accounts for 90% of peroxisome biogenesis. PEX11B functions as a key regulator in peroxisome fission, playing a vital role in membrane remodeling, deformation, and elongation [27, 52, 53]. PEX11B contains two membrane-spanning domains, with a short C-terminus and a larger N-terminus facing the cytosol [53]. Through bioinformatics analysis, potential palmitoylation sites on PEX11B have been identified (https://swisspalm.org/) (Fig. 5A). It is worth noting that proteomic analysis in adipocytes has identified PEX11B as a potential palmitoylated protein [54], prompting further investigation into its palmitoylation in SCs. The acyl-resin-assisted capture (acyl-RAC) assay confirmed that PEX11B can be palmitoylated (Fig. 5B). The oligomerization or self-interaction of PEX11B is crucial for its functional activity [53]. We discovered that PA disrupts PEX11B's self-interaction, while 2-BP can restore it (Fig. 5C, D), indicating that PA inhibits PEX11B's self-interaction through palmitoylation. The bioinformatics analysis has identified multiple potential palmitoylation sites on PEX11B (Fig. 5A), with many sites located at the C-terminal within the peroxisomal lumen, and specifically focusing on the highly conserved C18 and C25 sites in the N-terminus (Fig. 5E), indicating their likely significance as potential functional palmitoylation sites for PEX11B. To further determine the site of PEX11B palmitoylation, we mutated cysteine residues 18 and 25 to serine (S) to construct C18S and C25S mutant. We found that both mutants did not alter the expression level of the PEX11B protein (Supplemental Fig. 1E). Importantly, the mutation of C25S in PEX11B abrogated its palmitoylation, indicating that C25 is the major palmitoylation site of PEX11B (Fig. 5F). Additionally, the Acyl-Biotin Exchange (ABE) assay also yielded consistent results (Fig. 5G). We next investigated whether mutations in the palmitoylation site on PEX11B inhibit the effect of PA in reducing peroxisome abundance. PEX11B overexpression stimulates the generation of tubular peroxisomes or juxtaposed elongated peroxisomes (JEPs) [53, 55], consequently augmenting the peroxisomal abundance. Our investigations revealed that overexpression of the wild-type PEX11B leads to the formation of tubular membrane extensions by pre-existing peroxisomes, specifically those identified by PMP70 (denoted by white arrows); Notably, PA was capable of inhibiting this promotional effect (Fig. 5H, I). Conversely, PA was ineffective in attenuating the tubular membrane extensions induced by the PEX11B C25S mutant (Fig. 5H, I), indicating that mutation in the palmitoylation site on PEX11B inhibits the effect of PA in reducing peroxisome biogenesis. Taken together, these results indicate that PA-induced palmitoylation of PEX11B regulates peroxisome biogenesis.

### The PPAR agonist fenofibrate rescues PA-induced peroxisomal defects and restore the peroxisomal abundance in diabetic mice

**Fig. 6:**
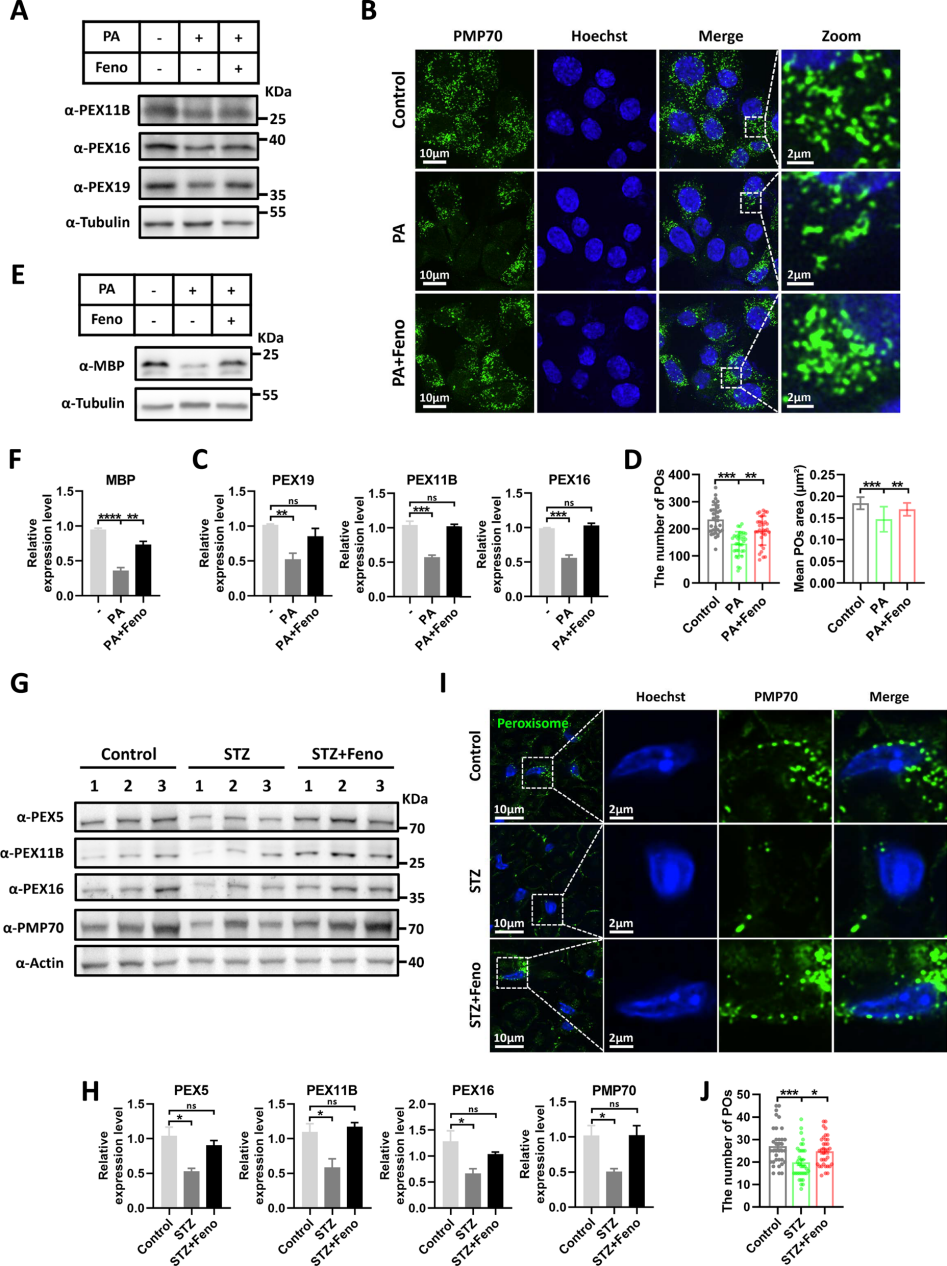
Fenofibrate, a PPAR agonist, reverses the decrease in peroxisomal abundance and restores the peroxisomal abundance in diabetic mice. **A** Western blot detection of peroxisomes-related proteins under PA (400 μM) and PA (400 μM) with Fenofibrate (Feno) (50 μM) conditions for 24 h in RSC96 cells. Tubulin was used as a loading control. **B** Representative immunofluorescence staining for PMP70 (green) under PA (400 μM) and PA (400 μM) with Fenofibrate (Feno) (50 μM) conditions for 24 h in RSC96 cells. Zoom: enlarged image of area indicated by dashed box. **C** Quantification of peroxisomes-related proteins. Data is shown as means ± SEM with One-way ANOVA Tukey's test (n = 3 independent experiments). ns, no significance. **p < 0.01, ***p < 0.001. **D** Quantification of peroxisomes (POs) area and number. Data is shown as means ± SD with One-way ANOVA Dunnett’s test (n = 32 cells). **p < 0.01, ***p < 0.001. **E** Western blot detection of MBP protein under PA (400 μM) and PA (400 μM) with Fenofibrate (Feno) (50 μM) conditions for 24 h in RSC96 cells. Tubulin was used as a loading control. **F** Quantification of MBP protein. Data is shown as means ± SEM with One-way ANOVA Tukey’s test (n = 3 independent experiments). ns, no significance. **p < 0.01, ***p < 0.001. **G** Immunoblotting with antibodies against peroxisomes-related proteins in sciatic nerves (n = 3 per group). **H** Quantification of peroxisomes-related proteins. Data is shown as means ± SEM with One-way ANOVA Dunnett's test. ns, no significance. *p < 0.05. **I** Representative immunofluorescence staining for PMP70 (green) on sciatic nerves sections. Right: enlarged image of area indicated by dashed box. **J** Quantification of peroxisomes (POs) number. Data is shown as means ± SD with One-way ANOVA Dunnett’s test (n = 35 cells). *p < 0.05, ***p < 0.001

Fenofibrate, belonging to the fibrate class and acting as a PPARα agonist, is commonly used in clinical settings for the treatment of hyperlipidemia [56, 57]. Previous research has revealed its ability to enhance peroxisome biogenesis [58, 59]. Therefore, we aimed to investigate whether fenofibrate can restore peroxisomal dysfunction in PA-treated SCs. In experiments conducted under PA treatment conditions with the addition of fenofibrate, the results showed that fenofibrate can rescue the levels of peroxisomal proteins (Fig. 6A, C). Consistently, confocal microscopy experiments demonstrated that fenofibrate also rescues the abundance of peroxisomes (Fig. 6B, D). Myelin basic protein (MBP) is a major protein component of myelin, accounting for approximately 30% of its composition [60, 61]. Importantly, we observed that PA significantly reduced the expression levels of MBP, while fenofibrate can restore the levels of MBP protein (Fig. 6E, F). Additionally, we also examined whether fenofibrate affects the PEX11B palmitoylation level. Our experiments showed that fenofibrate can reduce PA-induced PEX11B palmitoylation (Supplemental Fig. 1F, G). We next investigated whether fenofibrate restores the abundance of peroxisomes in diabetic mice. We found that fenofibrate intervention can rescue the expression levels of peroxisome protein (Fig. 6G, H). Furthermore, using super-resolution imaging, we also observed a rescue in the number of peroxisomes by fenofibrate intervention (Fig. 6I, J). These findings indicate that fenofibrate faithfully rescued peroxisomal dysfunction induced by PA and restored peroxisomal abundance in diabetic mice.

### A significant causal effect of diabetic neuropathy on multiple sclerosis

**Fig. 7:**
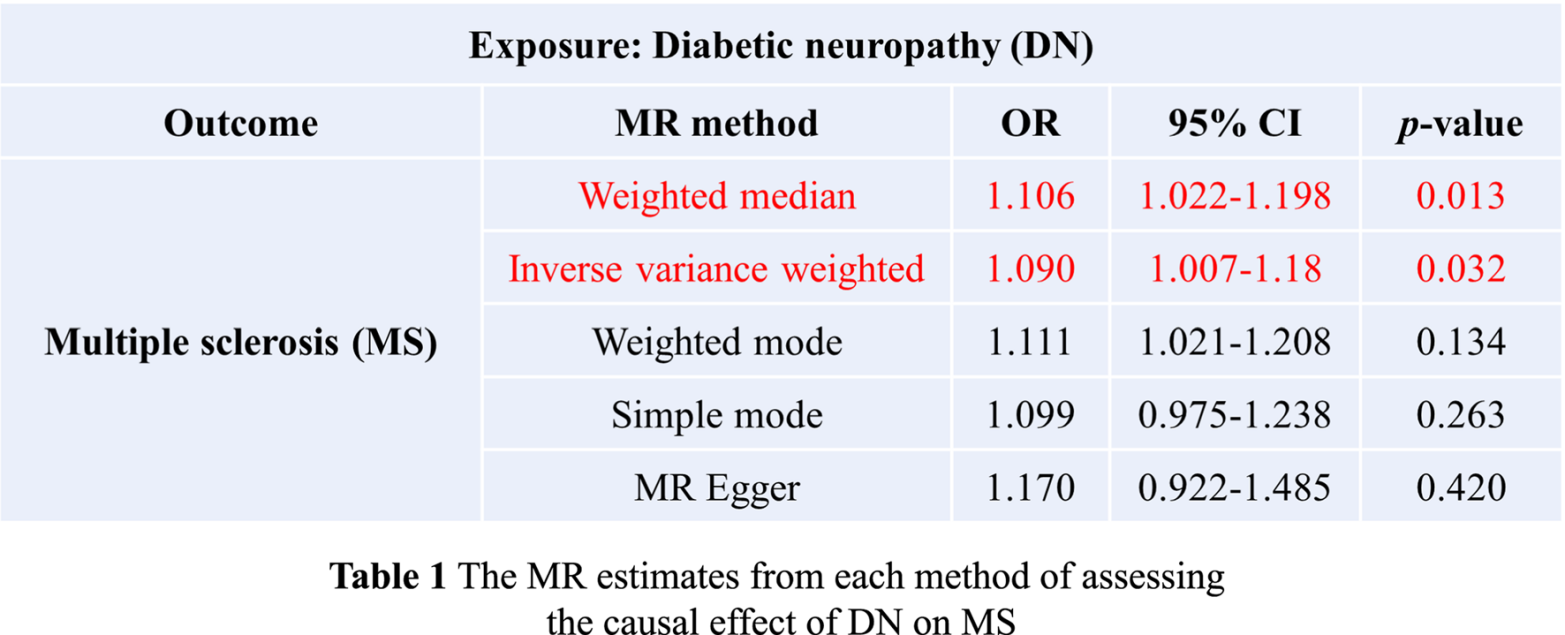
The MR estimates from each method of assessing the causal effect of DN on MS

Mendelian randomization (MR) is an important causal inference method that utilizes genetic variations as instrumental variables to overcome challenges in data acquisition and poor external validity in traditional epidemiological studies [62, 63]. Epidemiological evidence has shown a close association between diabetes and multiple sclerosis (MS) [64], a neurological immune disorder characterized by demyelination [65]. Importantly, peroxisomes dysfunction can lead to demyelinating diseases like MS [29]. To further validate the role of peroxisomes dysfunction in the pathogenesis of DN, we propose a hypothesis suggesting a causal relationship between DN and MS, indicating that DN may increase the risk of developing MS. Through MR analysis, we have identified a significant association between DN and an increased risk of MS in European populations. Specifically, for every 1 standard deviation (SD) increase in DN predisposition, the odds ratio (OR) for MS was estimated to be 1.09 [IVW; 95% CI 1.01–1.18; *p* = 0.03] and 1.11 [WM; 95% CI 1.02–1.20; *p* = 0.01] (Fig. 7) (Supplemental Figs. 2 and 3), suggesting a causal relationship where higher DN levels are associated with an elevated risk of MS. The MR-Egger test did not detect any evidence of directional pleiotropy (Egger intercept = − 0.04, *p* = 0.64), further confirming the results. Additionally, there was no significant heterogeneity observed for the IVW method (Cochran Q statistic: *Q* = 2.34, *p* = 0.31) and MR-Egger method (Cochran Q statistic: *Q* = 1.67, *p* = 0.2). Additionally, we found no evidence of causal relationship in the reverse direction between DN and MS due to the presence of directional pleiotropy (Egger intercept = − 0.08, *p* = 0.007). The results above indicate a significant causal effect of DN on MS, highlighting the importance of preventing MS at the onset of DN.

## Discussion

**Fig. 8:**
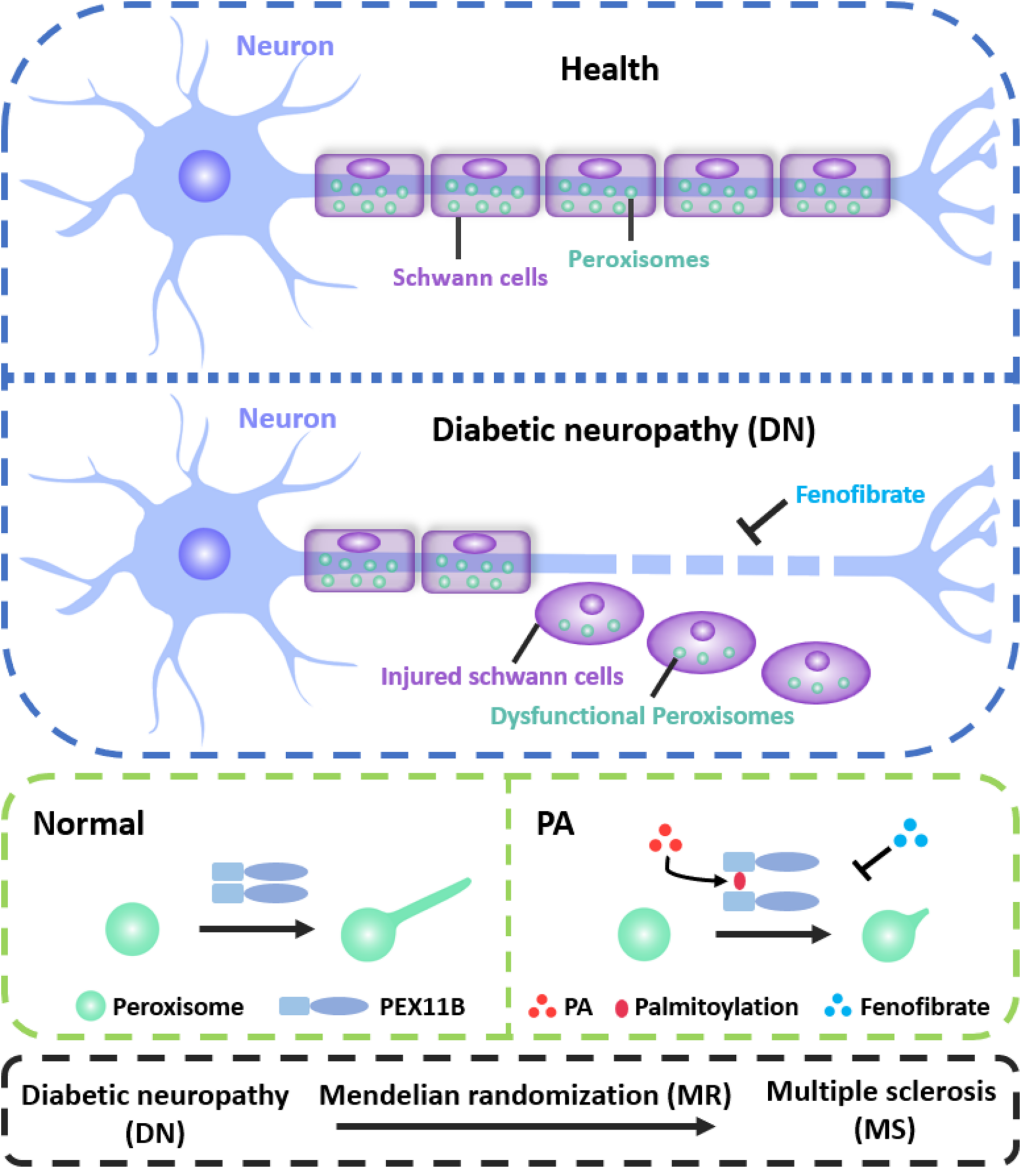
Schematic diagram showing the peroxisomal dysfunction in DN. At the animal level, a decrease in peroxisomal abundance was observed in the sciatic nerves of diabetic mice; Fenofibrate treatment rescued peroxisomal dysfunction in these mice. At the cellular level, PA treatment inhibits peroxisomal biogenesis; Mechanistically, PA treatment led to the palmitoylation of PEX11B, thereby disrupting its self-interaction and inhibiting peroxisomal elongation. Additionally, Fenofibrate treatment could restore peroxisomal abundance. MR analysis indicated a significant causal influence of DN on MS

In this study, we revealed a reduction in peroxisomal abundance in the sciatic nerve of DN mice and peroxisomal dysfunction induced by PA in SCs. These findings suggest that peroxisomal dysfunction may play a significant role in the pathogenesis of DN. We further elucidated the molecular mechanism underlying peroxisomal dysfunction, revealing that palmitoylation regulates peroxisomal abundance and demonstrated PEX11B undergoes palmitoylation, which impairs its self-interaction. Additionally, we identified the palmitoylation site (C25) on the PEX11B protein and demonstrated that mutation at this site confers resistance to the effect of PA in regulating peroxisome biogenesis. Importantly, our study also revealed that fenofibrate can restore peroxisomes abundance in diabetic mice. Lastly, MR analysis indicates a significant causal effect of DN on MS (Fig. 8). Overall, these results unveil a novel pathogenic mechanism of DN and underscore the potential of fenofibrate as a preferred treatment for preventing and treating DN.

Peroxisomes are dynamic organelles that interact with other metabolic organelles, such as lipid droplets and mitochondria, to play unique roles in lipid metabolism [23, 27, 66]. They are highly dynamic and regulated by active processes that control peroxisome abundance, such as peroxisome fission and deradation. Peroxisomal dysfunction is closely associated with the development of obesity, diabetes, and neurodegenerative disorders [26]. Therefore, maintaining a balance between peroxisomal biogenesis and degradation is essential for normal physiological function. Our findings showed a significant decrease in peroxisomal abundance in the sciatic nerve of diabetes mice (Fig. 1), indicating that peroxisomal dysfunction may be a key pathological feature of DN. It is important to note that the main clinical pathological feature of DN is demyelination [3, 4], which is consistent with the neuropathological features of peroxisomal diseases. Recent research has indicated that peroxisome biogenesis is dysregulated under insulin resistance [67]. Consistent with this, we observed that PA induces a decrease in peroxisomal abundance in SCs (Fig. 2). Further investigation revealed that PA did not affect pexophagy before the decrease in peroxisomes abundance (Supplemental Fig. 1A–D). Additionally, we found that Long-time PA exposure could prevent pexophagy, which may exacerbate peroxisomal dysfunction (Fig. 3), due to the downregulation of multiple pexophagy receptors such as PEX5 and PMP70 [48, 55]. These results suggest that PA primarily reduces peroxisomal abundance by impairing peroxisomal biogenesis.

Palmitoylation is a reversible posttranslational modification of proteins in which palmitates covalently bind to cysteine residues mostly via thioester linkages [32]. Dysregulation of protein palmitoylation has been closely linked to neurodegenerative diseases, inflammatory diseases, and cancer [32, 68]. Recent research has also revealed that palmitoylation is involved in diabetes and its complications [31, 38]. For example, increased NLRP3 palmitoylation has been associated with triggering proinflammatory macrophage phenotypes and pathological inflammation in diabetic wound healing [38]. Our study revealed that palmitoylation regulates peroxisome abundance in SCs and revealed PEX11B undergoes palmitoylation (Figs. 4 and 5). Functionally, palmitoylation can regulate protein membrane anchoring, trafficking, and interactions [32]. We further found that palmitoylation of PEX11B inhibits its self-interaction (Fig. 5), thereby hindering peroxisome fission. These findings support the idea that PA-induced lipotoxicity increases PEX11B palmitoylation, impairs peroxisomal function, and ultimately contributes to the development of DN. Further research on the specific DHHC proteins involved in regulating PEX11B palmitoylation could indeed lead to the discovery of novel therapeutic targets for the treatment of diabetic peripheral neuropathy.

Additionally, fenofibrate has been shown to reduce the global vascular risk in type 2 diabetes patients and has an impact on atherogenic dyslipidemia associated with the metabolic syndrome [69]. It also increases the levels of very-long-chain sphingolipids and exerts anti-inflammatory and anti-apoptotic actions in the endocrine pancreas [70]. These properties make fenofibrate a potentially promising treatment option for various complications related to diabetes and metabolic syndrome. It’s worth noting that pioglitazone, a member of the thiazolidinedione class and a ligand for PPARγ, has been recognized for its beneficial effects on macrovascular complications in diabetes and has shown promise in experimental diabetic neuropathy [71]. Our study indicates that fenofibrate has the potential to rescue peroxisomal dysfunction induced by PA and restore the peroxisomal abundance in diabetic mice (Fig. 6). This suggests that fenofibrate may exert a protective effect on peroxisomes. Moreover, our MR analysis indicates a causal relationship between DN and MS, with DN increasing the risk of developing MS. This indirectly corroborates our findings that peroxisomal dysfunction is a significant factor in the occurrence and progression of DN. Demyelination is indeed a key pathological feature of MS. Targeting peroxisomes could also represent a pivotal strategy in MS treatment, thereby suggesting the potential therapeutic utility of fenofibrate. These discoveries warrant further comprehensive exploration.

## Conclusions

Our study highlights peroxisomal dysfunction as a potential key pathogenic mechanism in DN, with palmitoylation playing a regulatory role in peroxisome biogenesis. Additionally, we have demonstrated a significant causal effect of DN on MS, underscoring the importance of preventing MS at the onset of DN. As a result, targeting peroxisomes may represent a novel strategy for preventing and treating DN, with fenofibrate emerging as a promising drug for DN.

## Supplementary Information

Additional file 1.

## Data Availability

Not applicable.

## Abbreviations

DN: Diabetic neuropathy
MS: Multiple sclerosis
PBDs: Peroxisomal biogenesis disorders
SCs: Schwann cells
POs: Peroxisomes
LDs: Lipid droplets
PA: Palmitic acid
MR: Mendelian randomization
APTs: Acyl-protein thioesterases
PPAR: Peroxisome proliferator-activated receptor
MBP: Myelin basic protein
