# Supplementary material for: PEX11B palmitoylation couples peroxisomal dysfunction with Schwann cells fail in diabetic neuropathy
Source: J Biomed Sci. 2025 Feb 12;32:20. doi: 10.1186/s12929-024-01115-5 (PMC11818136; doi:10.1186/s12929-024-01115-5)
Supplement: Supplementary file 1 — Additional file 1. [file 12929_2024_1115_MOESM1_ESM.pdf]

Supplemental Figure 1

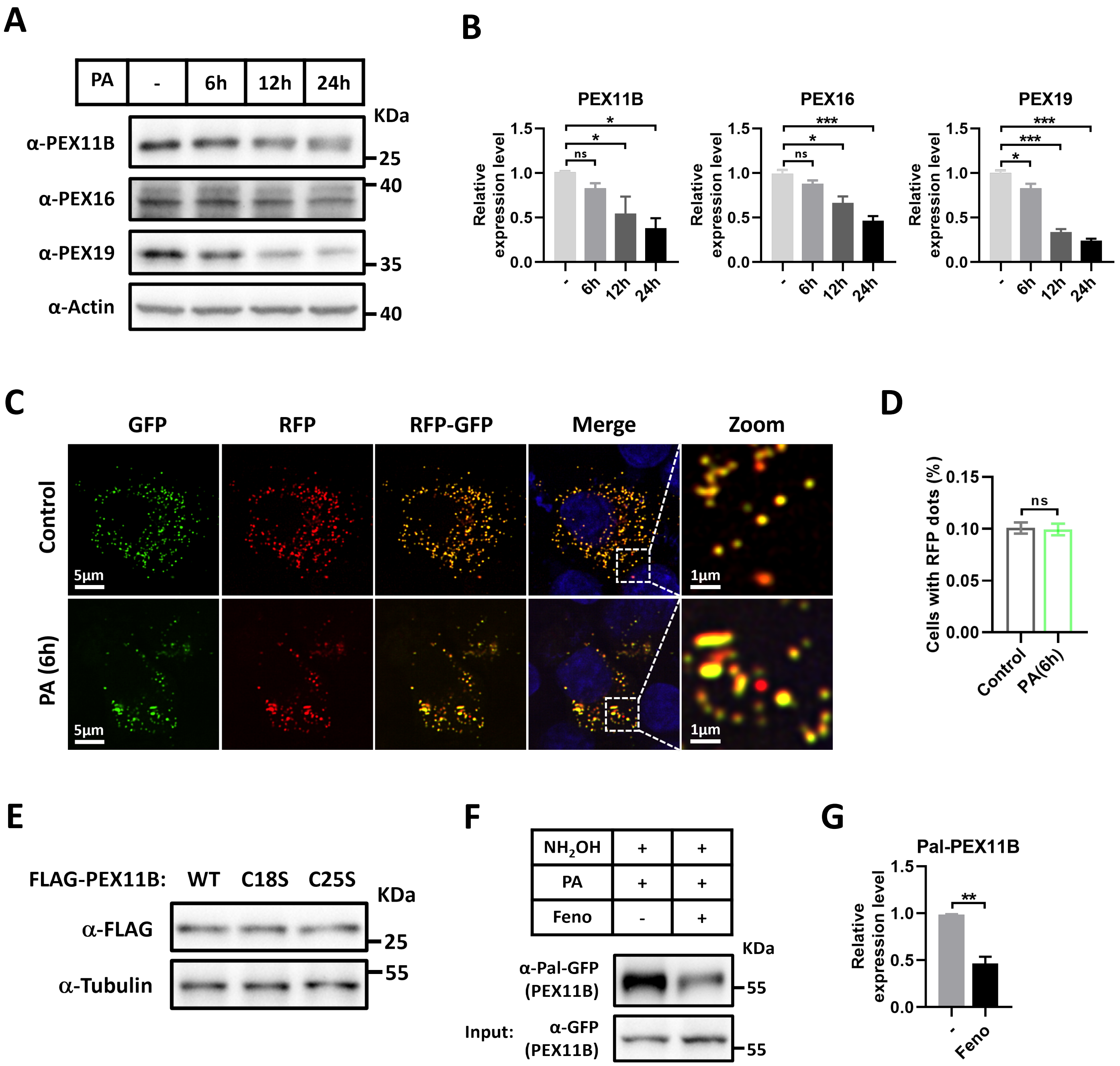

Supplemental Figure 2

A

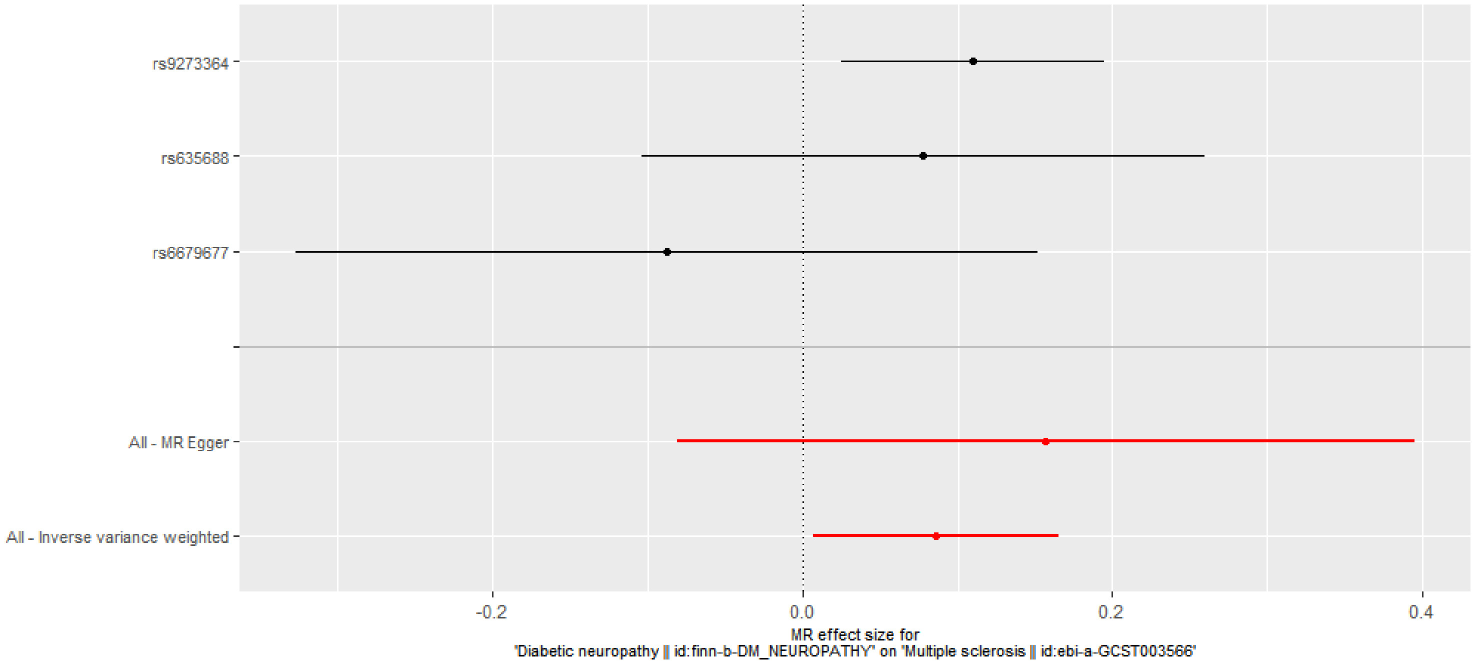

Forest plot of the two-sample Mendelian randomization analysis

B

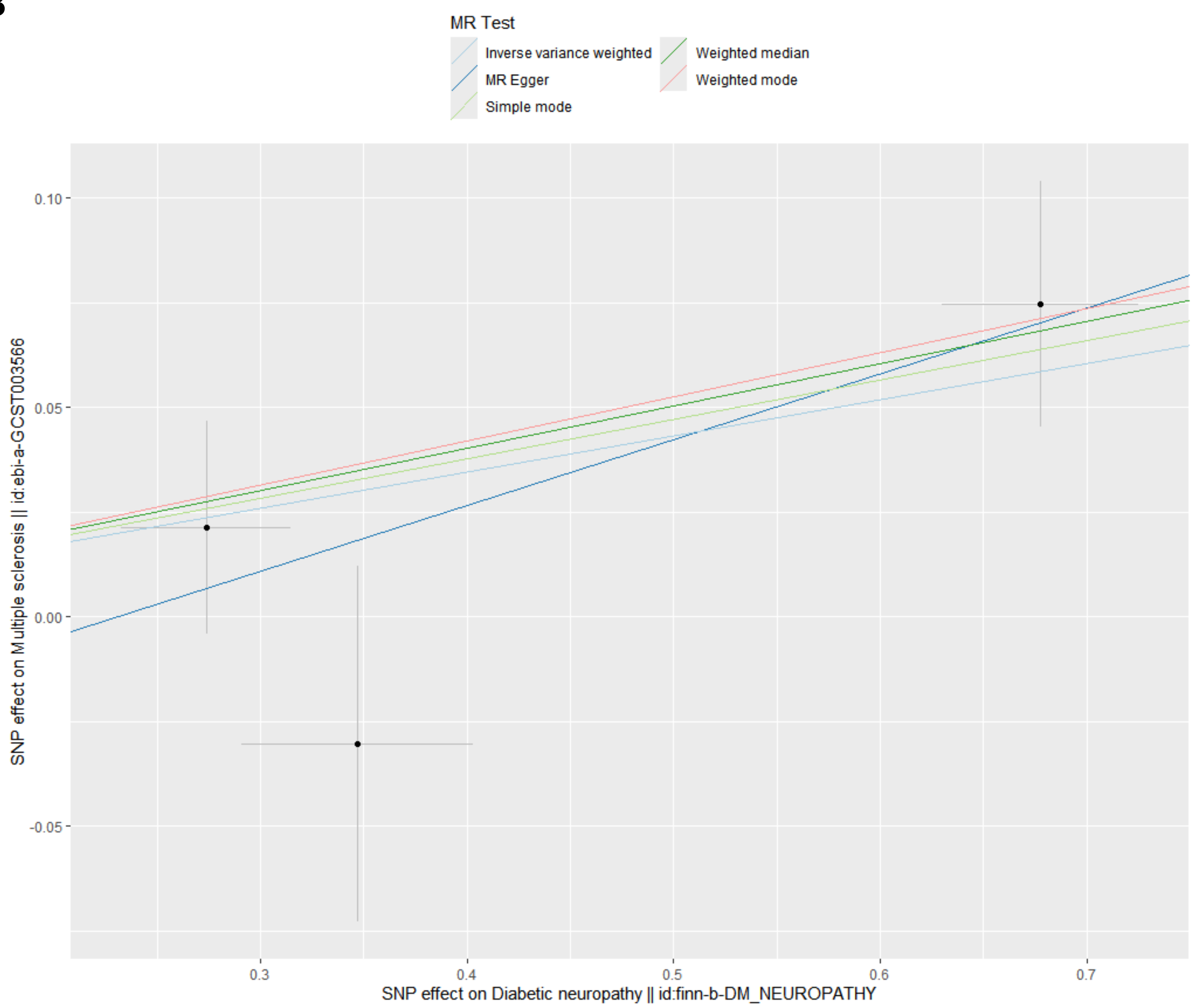

Scatter plot of the two-sample Mendelian randomization analysis

Supplemental Figure 3

A

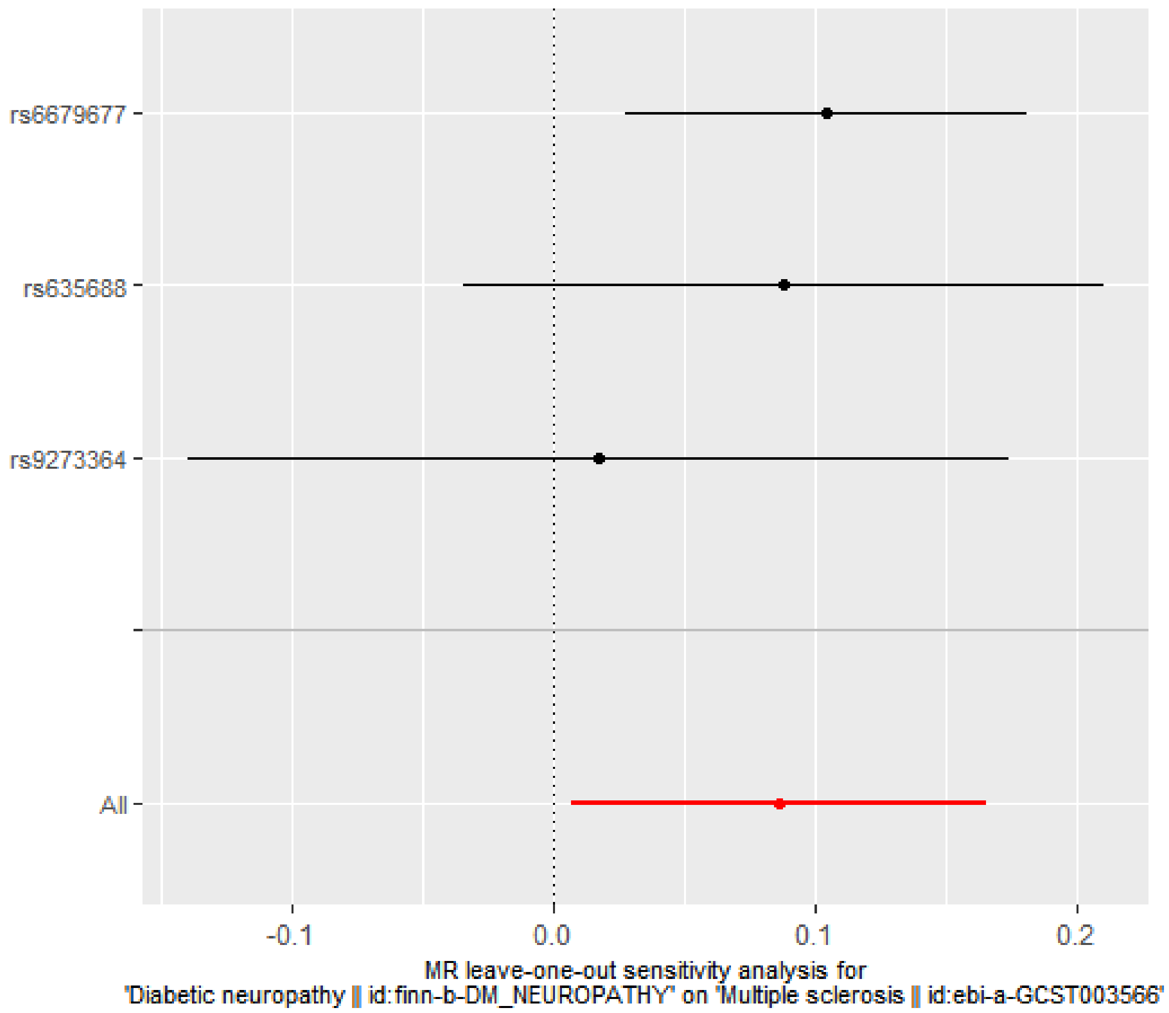

Outcome of the "leave-one-out" sensitivity analysis

B

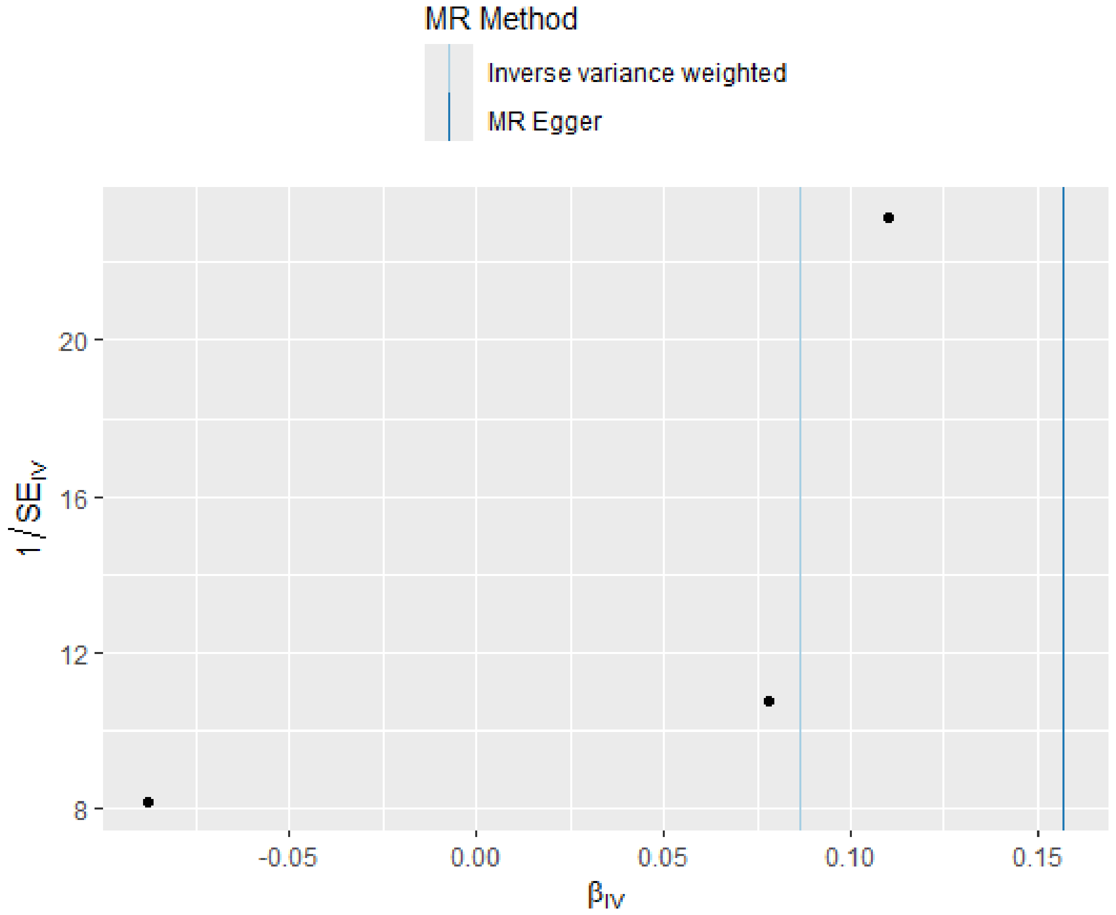

Funnel plot assessing heterogeneity
